# Northward expansion of the thermal limit for the tick *Ixodes ricinus* over the past 40 years

**DOI:** 10.1186/s13071-025-07084-4

**Published:** 2025-11-05

**Authors:** Daniele Da Re, Gaëlle F. Gilson, Quentin Dalaiden, Hugues Goosse, René Bødker, Lene Jung Kjær, Nicholas H. Ogden, Roberto Rosà, Sophie O. Vanwambeke

**Affiliations:** 1 Center for Earth and Climate Research, Earth & Life Institute, Université catholique de Louvain (UCLouvain), Louvain-La-Neuve, Belgium; 2 Center Agriculture Food Environment, University of Trento, San Michele All’ Adige, Trento, Italy; 3 Research and Innovation Centre, Fondazione Edmund Mach, San Michele All’Adige, Trento, Italy; 4 Nansen Environmental and Remote Sensing Center and Bjerknes Center for Climate Research, Bergen, Norway; 5 Department of Veterinary and Animal Sciences, Faculty of Health and Medical Sciences, University of Copenhagen, Copenhagen, Denmark; 6 Modelling Hub Division, Science and Policy Integration Branch, Public Health Agency of Canada, Ottawa, Canada; 7 Groupe de Recherche en Épidémiologie Des Zoonoses Et Santé Publique, Faculté de Médecine Vétérinaire, Université de Montréal, St-Hyacinthe, Montreal, QC Canada; 8 Centre de Recherche en Santé Publique, Université de Montréal, Montreal, QC Canada

**Keywords:** *Ixodes ricinus*, Climate change, Degree days, Hindcast, Scandinavia

## Abstract

**Background:**

The sheep tick *Ixodes ricinus* is the vector associated with the highest incidence of vector-borne disease in humans in Europe. Several studies have been published about the effect of future climate change on the potential distribution of *I. ricinus*, despite a limited understanding of how climate change has resulted in distribution changes to date. The objective of the present study was to assess whether temperature changes have already influenced the northern distribution limit of *I. ricinus* in Europe. To this end, we estimated a thermal threshold for the presence of the species and then used this estimated threshold to hindcast the geographical location of the thermal limit over the past 40 years.

**Methods:**

We used a public dataset of *I. ricinus* abundance at the northern edge of its European distribution for 2016–2017 and temperature data obtained from the ERA5Land dataset to identify a thermal threshold for *I. ricinus* distribution. We first modelled nymphal tick abundance as a function of cumulative annual degree days (ADD) > 0 °C stratified by biogeographical regions using observations for 2016–2017. We then identified the thermal limit for each biogeographical region as the minimum DD > 0 °C value where the predicted nymph abundance is greater than zero and projected it onto ERA5Land temperature data for the period 1979–2020.

**Results:**

Hindcasting the identified thermal limit suggested that *I. ricinus* has expanded its range by approximately 400 km in the Boreal biogeographical region between 1979 and 2020. This finding helps explain numerous observations of *I. ricinus* in areas presumed to be newly colonised.

**Conclusions:**

Our findings suggest a substantial northward expansion of *I. ricinus* over the past four decades. Our approach appears promising for understanding species distribution changes driven by recent climate change, acknowledging that multiple other factors affect tick distribution and abundance at the local scale, such as host distribution and microhabitat. Our results underline the relevance of long-term time series data and the risk associated with short time series for observing changes in distribution.

**Graphical abstract:**

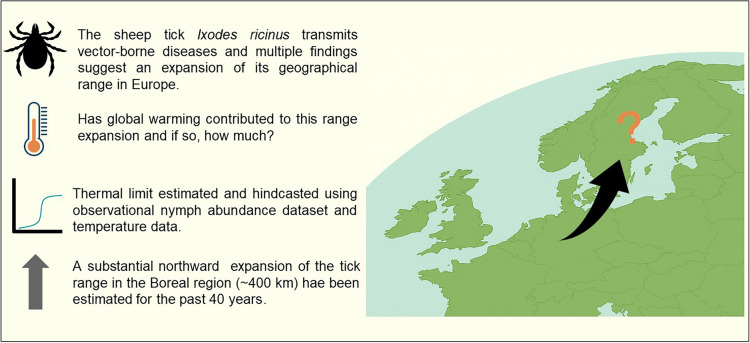

**Supplementary Information:**

The online version contains supplementary material available at 10.1186/s13071-025-07084-4.

## Background

The effect of climate change on the distribution and abundance of arthropod pathogen vectors is expected to be significant, but its importance relative to other environmental and societal factors has been much debated for decades. Arthropod vector development, activity and survival are regulated by temperature [1], hence the potential effect of climate change on ticks has generated many studies (reviewed in [2]). Climate change as a driver of geographic range expansion of ticks has drawn attention of researchers throughout the northern hemisphere, where ticks are of public health concern [3–6]. As the climate of Europe's northern regions has been warming faster than the change in global mean temperature, and is projected to maintain this trend in the coming decades, this temperature increase has already been identified as an important factor influencing the northward expansion and range shifts of various species [7–9]. The range of *Ixodes* spp. ticks in northern latitudes and high altitudes is thus expected to expand in relation to the limiting effects of temperature.

*Ixodes ricinus* is the most important pathogen vector in Europe, transmitting viral, protist and bacterial pathogens to humans, livestock and companion animals [10]. *Ixodes ricinus* ticks spend most of their life off the host and, like all arthropods, are sensitive to climate-driven abiotic factors such as temperature and humidity/saturation deficit [11]. Temperature affects tick survival, interstadial development rates and activity, while humidity influences survival and activity [1, 2]. Temperature and humidity also indirectly affect tick survival by affecting the availability of resources, such as suitable habitat and host availability [12]. When weather conditions are unsuitable for host finding (heat, drought and cold), *I. ricinus* ticks interrupt activity and shelter [1, 13]. Because of their capacity to shelter and diapause when conditions are unfavourable [14], ticks may mostly be affected by long-term climate changes rather than short-term weather variations [15].

Shifts in altitudinal limits in tick distribution reported in Eastern Europe [16, 17] and the Alps [18, 19] provided early indications of the potential effects of climate change on *I. ricinus* distribution. Using a 35-year-long dataset in Russia, Korotkov et al. [20] demonstrated an increase in adult *I. ricinus* abundance that could be related to a lengthening of the tick activity season, as host abundance was stable. In Norway, substantial changes in an altitudinal gradient of tick abundance have been observed [21], as well as range spread along a latitudinal gradient [22, 23]. Despite challenges related to the multifactorial nature of tick habitat and the demonstration of absence, a consensus now exists that the distribution of *I. ricinus* has changed and that climate change is likely one factor driving it.

Many studies that investigated climate change have focused on producing projections into the future (e.g. [24–26]), using mostly correlative approaches, meaning that no proper external validation can be carried out. These studies are usually habitat suitability models, using correlative associations between field observations, gathered from datasets that are limited in terms of having small sample sizes or using presence-only data [25, 27, 28] and mostly abiotic variables. Long-term data series of *I. ricinus* abundance are particularly lacking in areas where the health concern they bring is an emerging one. Mechanistic approaches may have more explanatory potential [29], but they have not been used beyond the local scales to explore possible effects of past climate change [30]. Mechanistic models are also constrained by incomplete quantitative measures of life-cycle parameters (e.g. development rates) according to temperature. In this context, drawing conclusions on the effects of climate change is often qualitative, when based on sets of heterogeneous data (e.g. [22]), or indirect, by using proxy indicators such as tick-borne pathogen data in humans or animals (e.g. [31]). For the latter, diagnostic capacity, reporting practices and disease knowledge may have changed over the years. Disease records for humans and livestock [31] show that the reported incidence of *I. ricinus* has increased during the period 1995–2015 and that climate has probably played a role, likely through effects on the vector, although effects of changing reservoir host dynamics cannot be ruled out. Assessing the role of climate change requires considering the very multifactorial nature of zoonotic disease incidence and how our capacity to monitor changes is affected by it. When focusing on the effects of climate on infected tick abundance, overall tick abundance and human disease incidence, respectively are partial views; while both are affected by societal factors generally unrelated to tick ecology, the latter is also affected by exposure and by reporting practices. Moreover, even though human cases only occur if infectious vectors are present, they represent a more visible, but often partial, part of the zoonotic iceberg [32].

In general, there is a poor understanding of past distribution changes that may or may not have been associated with climate change. However, observations of the effects of climate change on various arthropod vectors in Europe, including ticks, are accumulating [33]. In this paper, we use empirical data to infer a minimum threshold of thermal suitability for *I. ricinus*, which allows us to draw a continent-wide contour line of thermal suitability. We then evaluate the changing position of the thermal suitability limit across a period of 40 years since 1979. We adopt the use of cumulative annual degree-days above 0 °C (ADD > 0 °C) to define the thermal suitability threshold for *I. ricinus*, following the approach used by Ogden et al. to successfully define climatic suitability for *Ixodes scapularis* in Canada [34]. In that study, a temperature-driven population model showed that equilibrium tick abundance was strongly associated with ADD > 0 °C values (*r*^2^ > 0.96), which served as a reliable, mappable index of thermal conditions supporting tick persistence and seasonal activity. Ogden et al.'s model further indicated that tick survival declined progressively with lower temperatures, rather than being linked to a specific phenological threshold, making ADD > 0 °C a robust predictor of climatic constraints on tick distribution.

## Methods

We assume that tick population survival is primarily influenced by how long it takes ticks to fulfil their life-cycle, which will be strongly affected by interstadial development rates, which cover blood meal digestion, moulting and inactivity in unsuitable questing conditions [1]. During those periods, ticks make use of the refuges offered by tick-suitable habitats that shield ticks from extreme temperatures. Average daily temperatures > 0 °C are relevant for tick development and population survival, as demonstrated previously in a population model [34] calibrated on field and laboratory data on the effects of temperature on tick development, activity and survival (reviewed in [15]). While refuges allow ticks to survive freezing temperatures, and questing occurs at temperatures > 5 °C, Ogden et al. [34] demonstrated that ADD > 0 °C captures suitable temperature conditions as a summary measure. Although these studies focused on *I. scapularis*, considering the high ecological and entomological similarity between *I. scapularis* and *I. ricinus* [35], we consider it a relevant approach for assessing climate suitability for *I. ricinus* populations. In our study, suitable temperature conditions for *I. ricinus* population persistence are those above a critical temperature threshold needed for tick development and activity to allow the completion of the tick life-cycle. Below this threshold, conditions prevent a mated adult female from producing at least one surviving mated adult female, thus inhibiting population persistence.

**Fig. 1:**
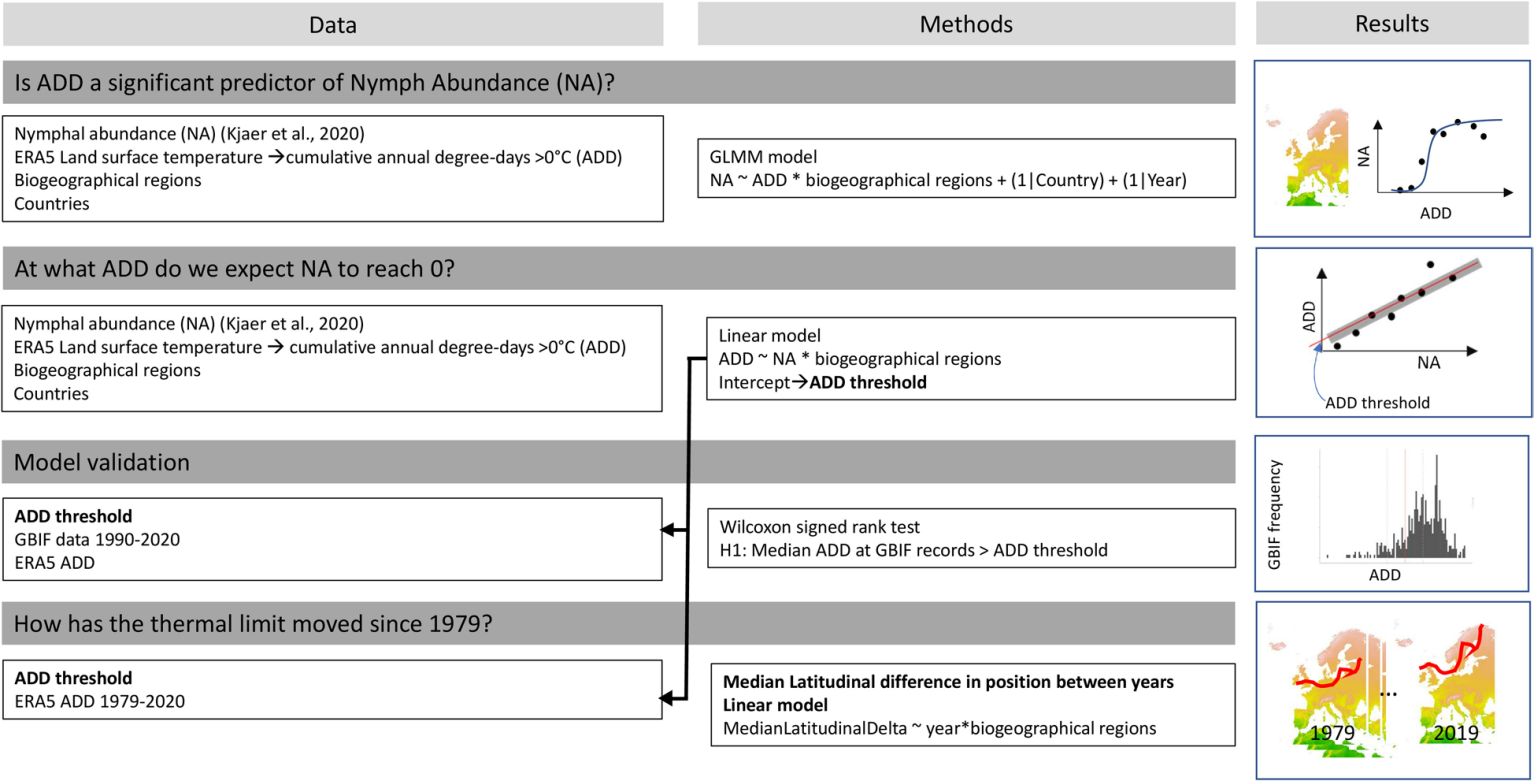
Summary of the methodology applied to investigate the relationship between tick nymph abundance and cumulative annual degree days > 0 °C (ADD > 0 °C), and the ADD thresholds for tick persistence in each biogeographical region. ADD, Annual degree days; ERA5, fifth generation European Centre for Medium-Range Weather Forecasts (ECMWF) atmospheric reanalysis; GBIF, Global Biodiversity Information Facility network; GLMM, generalised linear mixed model

Using a set of nymph count samples, we first assessed the effect of annual DD > 0 °C on nymph abundance using a generalised linear mixed model (GLMM). Having found a significant and positive association between annual DD > 0 °C and nymph abundance, we subsequently determined the minimum annual DD > 0 °C threshold at which nymph abundance is zero. This indicates the critical temperature threshold below which conditions are unfavourable for *I. ricinus* population persistence, and was set at the intercept of the relationships between annual DD > 0 °C and nymph abundance. We then mapped these thresholds over the area of interest and assessed their latitudinal changes over the period 1979–2020. An overview of the methods is presented in Fig. 1.

### Biological observations and area of interest

We used *I. ricinus* nymph counts acquired by dragging a white 1.05 × 1.15-m flannel cloth along two perpendicular 100-m transects, sampled both ways, as part of a survey conducted by Kjær et al. [36] across Denmark, southern Norway and south-eastern Sweden. Sites had to consist of 80% forest and 20% meadows in their study, and the authors gathered data on tick larval, nymph and adult abundance at 159 sites in south Scandinavia during August–September 2016, with an additional sampling of 30 sites surveyed during the same months in 2017, among which 18 sites were sampled both years[36]. Although known not to capture all individuals and to be affected by vegetation structure, field surveillance by dragging is considered the gold-standard method for identifying the presence of reproducing, self-sustaining tick populations [37, 38], and field sites were selected based on being similarly suitable for flagging.

**Fig. 2:**
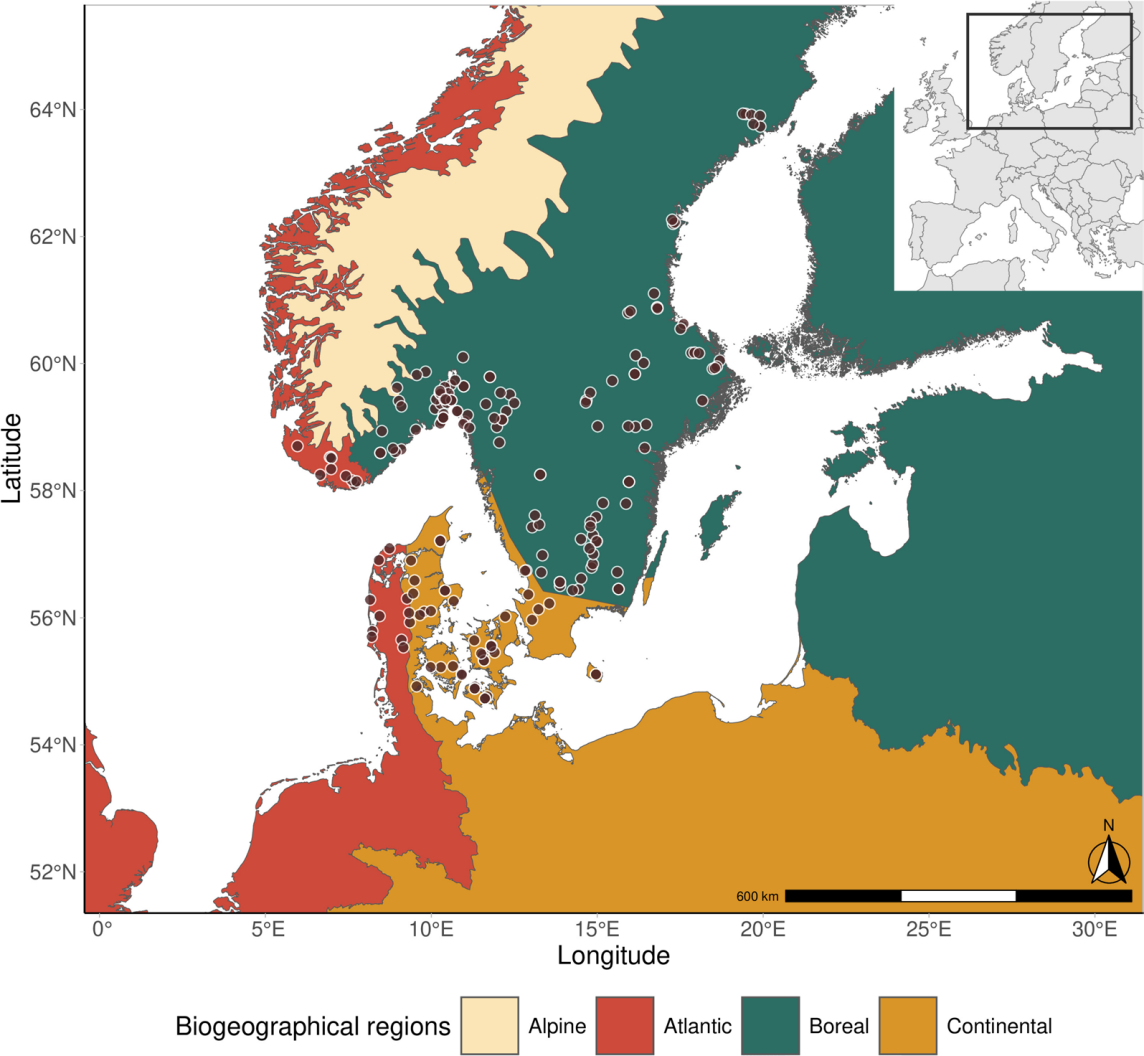
Biogeographical regions of Europe in the area of interest and the locations (brown dots) sampled in 2016–2017. The sampling locations broken down by year are available in Additional file 1: Figure S1

Sampling sites span from 5° to 20° East and from 54° to 64° North (Fig. 2). We used sampling sites located in three biogeographical regions, defined as Atlantic, Boreal and Continental (Fig. 2, according to [39]). We limited the geographical area of extrapolation of the model inferring the annual DD > 0 °C threshold to these biogeographical regions only.

### Environmental covariates

We calculated the annual DD > 0 °C, defined as the daily average number of degrees centigrade above 0 °C summed over a year (ADD > 0 °C) from 1979 to 2020 using the daily average surface temperature estimates at 9 × 9-km spatial resolution from the land reanalysis ERA5Land [40]. ERA5Land is a climate reanalysis dataset developed by the European Centre for Medium-Range Weather Forecasts (ECMWF), which combines historical observations with modern weather forecasting models to produce consistent, gridded estimates of past atmospheric and surface conditions worldwide.

### Effect of ADD > 0°C on nymph abundance

We used GLMMs to assess the dependence of nymph abundance sampled in 2016–2017 on temperature of the same years, using the interaction between ADD > 0 °C and biogeographical regions as fixed effects and country-level and year-level random effects included as grouping factors (Eq. 1).

1 $${\text{Nymph abundance}}\,\sim \,{\text{ADD}}\,> \,0 ^\circ {\text{C}} \times {\text{biogeographical regions}}\, + \,\left( {{1}|{\text{Country}}} \right)\, + \,\left( {{1}|{\text{Year}}} \right)$$

We conducted model selection through a stepwise comparison of alternative model structures. Model selection began with a full Poisson GLMM with quadratic effects on the ADD > 0 °C, its interaction with the biogeographical regions, and the inclusion of random effects to account for spatial and temporal structures. Including Country captures potential heterogeneity in sampling effort and habitat types, while Year accounts for inter-annual variability in tick population dynamics. Additionally, we tested overdispersion in the initial Poisson model and evaluated whether a negative binomial distribution would better capture the variance structure of the data. We also assessed the potential presence of zero inflation. Competing models were compared using Akaike Information Criterion (AIC) and likelihood ratio tests, with residual diagnostics performed using DHARMa (R package) simulations to evaluate model adequacy. Based on these comparisons, the final model described in Eq. 1 was selected with a negative binomial distribution. A full description of the model selection process is provided in Additional file 1 (S3-Description of the model selection). Summary statistics of the final model included the coefficient of determination (*R*^2^), the root mean square error (RMSE), and the mean absolute error (MAE). We also assessed the presence of spatial correlation in model residuals using Moran’s* I* statistic. All analyses were performed in R 4.4.0 [41], and the code used is available on GitHub at https://github.com/danddr/ticks_DD_Scandinavia.

### ADD > 0 °C threshold for *I. ricinus* population persistence

In the absence of substantial literature on the lower ADD > 0 °C limit for the persistence of *I. ricinus*, we defined the lower thermal limit for each biogeographical region as the intercept of the relationship between ADD > 0 °C and nymph abundance as measured by Kjær et al. [36] using a linear model (Eq. 2):

2 $${\text{ADD}}\,> \,0 ^\circ {\text{C}}\,\sim \,{\text{nymph abundance}} \times {\text{biogeographical regions}}$$

The intercept of the linear model represents the estimated value of ADD > 0 °C when nymph abundance is predicted to be zero.

To assess the estimated thresholds, we used an independent dataset of *I. ricinus* occurrences obtained from the Global Biodiversity Information Facility (GBIF) for the period 1979–2020 in our area of interest [42]. The occurrence data were filtered by removing missing or incorrect coordinates and occurrences located in the sea or the city centres of major cities. We subsequently linked each occurrence to its corresponding biogeographical region and annual ADD > 0 °C for the sampling year. Each occurrence was identified as below or above the threshold identified for that region. We defined GBIF occurrences above each threshold as ‘true positives’ and GBIF occurrences below each threshold as ‘false positives’ and computed sensitivity.

Additionally, we used a one-sample Wilcoxon signed-rank test to compare the median ADD > 0 °C values extracted from GBIF tick occurrence records, with the estimated thermal threshold derived from tick abundance data for each biogeographical region. The null hypothesis (H_0_) was that the median ADD > 0 °C at GBIF occurrence sites is equal to or less than the estimated threshold. The alternative hypothesis (H_1_) was that the observed median ADD > 0 °C is significantly greater than the threshold, suggesting that ticks are present in areas with higher heat accumulation than the minimum required for population persistence. We chose the Wilcoxon test because the distribution of the ADD > 0 °C obtained from the GBIF locations was non-normally distributed and subject to outliers, making a non-parametric test more appropriate than a standard t-test. The limitations of GBIF records, which result from passive, opportunistic sampling and vary in spatial and temporal resolution, preclude more structured statistical validation and introduce potential biases.

### Spatio-temporal trends in ADD > 0 °C thresholds

We computed the geographic position of the ADD > 0 °C threshold for each of the three sampled biogeographical regions for every year from 1979 to 2020. We then computed the latitudinal difference between the position of the threshold in each year compared to that in 1979 within each biogeographical region. We obtained a series of latitudinal difference values, from which we extracted the median latitudinal difference between 1979 and each yearly threshold. We then utilised linear regression to analyse the relationship between the median latitudinal difference between the position of the threshold in one specific year and 1979 (MedianLatitudinalDelta) (Eq. 3), with the aim to discern any systematic changes in the latitudinal range of thermal limits over the study period for different biogeographical regions.

3 $${\text{MedianLatitudinalDelta}}\,\sim \,{\text{year}} \times {\text{biogeographical regions}}$$

The threshold was mapped as isolines, connecting points of equal ADD > 0 °C value for 1979 and 2020.

## Results

The dataset from Kjær et al. [36] included 189 sampling events conducted in Denmark, Norway and Sweden, distributed across three biogeographical regions: Atlantic (*n* = 19), Boreal (*n* = 126) and Continental (*n* = 44). In 2016, most sampling in the Boreal region took place in Sweden (*n* = 70) and Norway (*n* = 40), while sampling at Continental sites occurred primarily in Denmark (*n* = 28) and Sweden (*n* = 5); Atlantic sites were sampled in Denmark (*n* = 9) and Norway (*n* = 7). In 2017, additional sampling occurred mainly in Sweden and Denmark, with boreal sites sampled in Sweden (*n* = 9) and Norway (*n* = 7); continental sites sampled in Denmark (*n* = 10) and Sweden (*n* = 1); and Atlantic sites sampled in Norway (*n *= 3; Additional file 1: Table S2).

A total of 1820 raw *I. ricinus* observations were obtained from the GBIF database in the area of interest, which were reduced to 836 observations after data cleaning. Following the exclusion of observations located in the alpine biogeographical region, the GBIF dataset was further refined to 809 observations, with 135 occurrences in the Atlantic, 547 in the Boreal and 127 in the Continental biogeographical regions.

The GLMM (Eq. 1) identified statistically significant associations between nymph abundance and both ADD > 0 °C and biogeographical regions (Table 1). In the Atlantic region, the reference category, ADD > 0 °C, showed a positive association with nymph abundance (estimate = 0.003, 95% confidence interval [CI] 0.001–0.005, *P* = 0.002), indicating that an increase of 1000 ADD > 0 °C corresponds to an expected increase of approximately 2.9 nymphs. A similar and stronger positive association was observed in the Boreal region (slope = 0.005, interaction estimate = 0.002, *P* = 0.032), but the Boreal region had a lower baseline nymph abundance than the Atlantic region (estimate = − 5.251, 95% CI − 11.209 to 0.708, *P* = 0.084). Baseline nymph abundance was higher in the Continental region than in the Atlantic (estimate = 7.041, *P* = 0.147). However, the interaction with ADD > 0 °C was negative (− 0.0019), which offsets the positive main effect. This resulted in a small net positive slope (approx. 0.001), although it was not statistically significant. A graphical representation of the estimated relationship between nymph abundance and ADD > 0 °C is displayed in Additional file 1: Figure S4 for each biogeographical region. Random effects analysis revealed substantial variation in nymph abundance across different countries (variance = 0.421, standard deviation [SD] 0.649) and years (variance = 0.208; SD 0.456), indicating that both spatial and temporal factors contribute to observed variation in tick abundance.

**Table 1:** Estimates of the fixed effects of the generalised linear mixed model showing the effects of annual degree days above 0 °C (ADD > 0 °C) and biogeographical regions on nymph abundance

Predictor	Estimate (95% CI)	SE	*z* value	*P* value
Intercept (Atlantic)	− 6.001 (− 11.599, − 0.403)	2.856	− 2.101	0.036
ADD > 0 °C	0.003 (0.001, 0.005)	0.001	3.101	0.002*
Boreal	− 5.251 (− 11.209, 0.708)	3.040	− 1.727	0.084*
Continental	7.041 (− 2.466, 16.548)	4.851	1.452	0.147
ADD > 0 °C:Boreal	0.002 (0.000, 0.004)	0.001	2.138	0.032*
ADD > 0 °C:Continental	− 0.002 (− 0.005, 0.001)	0.001	− 1.276	0.202

The reference level is the Atlantic biogeographical region

*ADD* Additional degree days,* CI* confidence interval,* SE* standard error

*Significant *P* value

The model performance metrics indicate that the conditional *R*^2^ value, the proportion of the variance explained by the fixed (ADD > 0 °C and biogeographical region) and random (country and year) effects, is 0.729. The marginal *R*^2^ value, representing the variance explained by fixed effects alone, is 0.546. Additionally, the RMSE and MAE are 41.314 and 25.277, respectively, expressed in the number of individual nymphs.

The linear regression model designed to identify the ADD > 0 °C threshold values (Eq. 2) achieved an *R*^2^ value of 0.565, with a significant intercept, which we interpreted as ADD > 0 °C thresholds below which tick persistence is not possible, in each biogeographical region (Table 2). The Continental biogeographical region showed the highest ADD > 0 °C threshold (3399 ADD > 0 °C), followed by the Atlantic (2847 ADD > 0 °C) and then by the Boreal biogeographical region, which showed the lowest threshold equal to 2682 ADD > 0 °C (Table 3).

**Table 2:** Linear model identifying the additional degree days > 0 °C threshold values; fixed effect estimates the tick nymph abundance and biogeographical regions on degree days above 0 °C (ADD > 0 °C)

Predictor	Estimate (95% CI)	SE	*z* value	*P* value
Intercept (Atlantic)	2847.436 (2701.880, 2992.993)	73.774	38.597	< 0.001*
Nymph abundance	6.995 (2.455, 11.535)	2.301	3.040	0.003*
Boreal	− 165.533 (− 321.067, − 9.999)	78.831	− 2.100	0.037*
Continental	551.841 (373.057, 730.624)	90.614	6.090	< 0.001*
Nymph abundance × Boreal	− 4.505 (− 9.255, 0.245)	2.407	− 1.871	0.063
Nymph abundance × Continental	− 6.705 (− 11.407, − 2.003)	2.383	− 2.814	0.005*

The reference level is the Atlantic biogeographical region

* CI* Confidence interval,* SE* standard error

*Significant *P* value

**Table 3:** Cumulative annual degree days > 0 °C thresholds for each biogeographical region and the sensitivity metrics for GBIF observations above (true positive) and below (false positive) the respective threshold

Biogeographical regions	ADD > 0 °C threshold (95% CI)	GBIF observations	GBIF ADD > 0 °C median	True positives	False positives	Sensitivity (± SE)	Wilcoxon test *P* value
Atlantic	2847 (2702, 2993)	135	2745	49	86	0.363 (± 0.041)	***P*** < 0.001*
Boreal	2682 (2381, 2983)	547	2997	455	93	0.832 (± 0.016)	***P*** < 0.001*
Continental	3399 (3075, 3724)	127	3460	76	51	0.598 (± 0.044)	0.586

*ADD* Additional degree days,* CI* confidence interval,* GBIF* Global Biodiversity Information Facility,* SE* standard error

*Significant *P* value according to the one-tailed Wilcoxon test

**Fig. 3:**
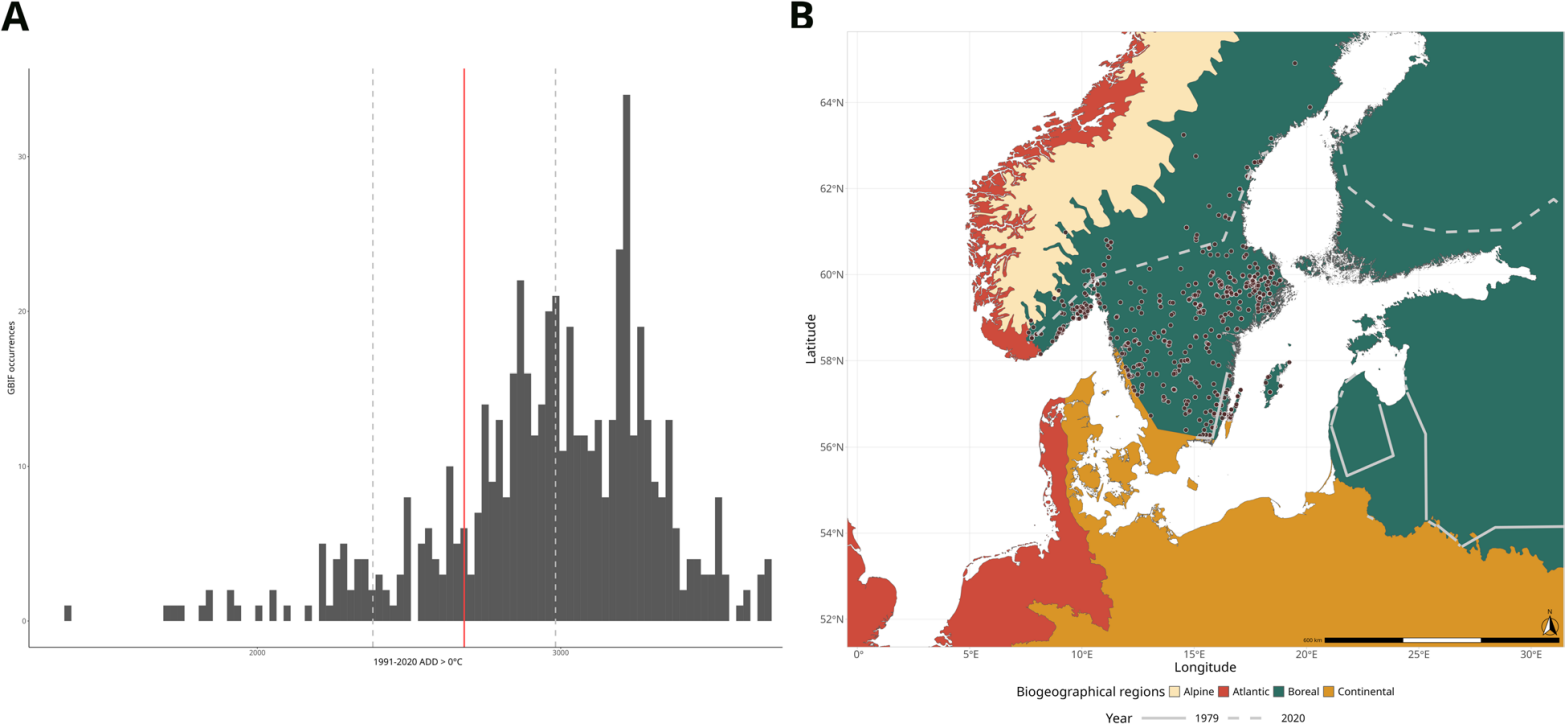
**A** Distribution of GBIF occurrence over ADD > 0 °C (histogram) and ADD > 0 °C threshold (red line; grey dashed lines represent the confidence interval) in the Boreal region.** B** Mapped ADD > 0° isoline threshold of the Boreal region for the reference years 1979 (solid line) and 2020 (dashed line). Brown dots represent the GBIF observations for the Boreal biogeographical region only. ADD, Annual degree days; GBIF, Global Biodiversity Information Facility network

The sensitivity metric based on the GBIF observations of *I. ricinus* scored 0.36 for the Atlantic region but was higher for the Boreal and Continental regions (0.83 and 0.60, respectively; Table 3, Fig. 3a; Additional file 1: Figure S5). The one-sample Wilcoxon signed-rank test indicated that the median of the GBIF-derived ADD > 0 °C values was significantly higher than the modelled threshold in both the Atlantic (*P* < 0.001) and Boreal (*P* < 0.001) regions, while no significant difference was detected in the Continental region (*P* = 0.586).

The model analysing the relationship between median latitudinal shift and year across biogeographical regions (Eq. 3) explained 39% of the variance (*R*^2^ = 0.39) and showed a significant positive effect of year (*P* = 0.003), indicating an overall northward shift of the thermal thresholds over time (Table 4). While the Atlantic region (reference category) showed a significant trend, interaction terms for the Boreal and Continental regions were not significant, suggesting similar rates of change across regions. This interpretation is supported by an analysis of variance (ANOVA), which confirmed a significant effect of year (*P* < 0.001), but no significant year × biogeographical region interaction (*P* = 0.16), indicating that the northward trend is consistent across biogeographical regions.

**Table 4:** Estimated temporal trends in latitudinal differences across the three biogeographical regions, with 95% confidence intervals

Predictor	Estimate (95% CI)	Std. Error	*z* value	*P* value
Intercept (Atlantic)	− 83.083 (− 138.176, − 27.989)	27.826	− 2.986	0.003*
Year	0.042/0.015, 0.070)	0.014	3.051	0.003*
Boreal	− 74.633 (− 152.548, 3.281)	39.352	− 1.897	0.060
Continental	− 35.611 (− 113.525, 42.304)	39.352	− 0.905	0.367
Year × boreal	0.038 (− 0.001, 0.077)	0.02	1.914	0.058
Year × continental	0.018 (− 0.021, 0.057)	0.02	0.921	0.359

*CI* Confidence interval,* SE* standard error

*Significant *P* value

We then focused only on the Boreal region as it had the threshold with the highest sensitivity values obtained using the GBIF data. Overall, in the period of interest, the Boreal ADD > 0 °C threshold moved north by 0.082 degrees of latitude/year (approx. 9 km; Fig. 3b), corresponding to a northward shift of approximately 400 km between 1979 and 2020. The threshold isolines for the other regions are presented in Additional file 1: Figures S5, S6.

## Discussion

In this study, we examined the link between nymphal abundance and annual DD > 0 °C. We projected this relation to temperature data spanning back to 1979 to explore the potential effect of temperature change on *I. ricinus* distribution. Using our biogeographical region-specific thresholds, we found that the thermal limit identified for the Boreal region, which covers the greatest extent of our study area, progressed northward. In 1979, thermal conditions were unsuitable throughout Norway, most of Sweden and Finland. In contrast, according to our study, conditions are now suitable in the Boreal region from the southeastern shore of Norway to the southern and central regions of Sweden and the south of Finland, suggesting an overall northward shift of the limiting conditions of tick persistence of approximately 400 km over the 1979–2020 period. We believe our estimate of range expansion is conservative compared to those reported in previous studies that based their conclusions on presence observations, as abundance allows the relationship with temperature to be distinguished with more confidence, even though we did not account for other dimensions of habitat such as microclimate, vegetation and hosts [43].

We assessed whether temperatures are permissive for *I. ricinus* persistence using ADD > 0 °C, a useful index for understanding the effect of temperature on the tick life-cycle ([34]; reviewed in Ebi et al. [44]). Temperatures > 0 °C determine tick development rates and thus the length of the tick life-cycle, with warmer temperatures accelerating (to a point) development [1], even though host-finding success and other factors, such as spring warming [45], may also affect the length of the life-cycle. When ticks develop very slowly, populations cannot persist, meaning that a temperature threshold can be identified below which populations cannot persist [35]. Annual cumulative degree days > 0 °C can thus be used to assess changes over time in temperature conditions for *I. ricinus* population persistence. We focused on nymphs as the primary indicator of tick population persistence whereas, in principle, only the presence of each life stage can identify an established population. However, larvae occur in clusters in association with the egg masses from which they hatched, and adults are much fewer due to interstadial mortality. Nymphs are generally considered to be of greater public health concern and often constitute the focus of field sampling, leading to our choice in the present study. Beyond field sampling practices and constraints, we believe nymphs to be a suitable indicator, especially considering that our method includes abundance and not solely presence. A more important caveat of our method may reside in the size of the dataset used to calibrate the models.

When fitting the GLMM model (Eq. 1), we identified statistically significant relationships between nymph abundance, ADD > 0 °C and biogeographical regions, with significant interactions between biogeographical regions and ADD > 0 °C. The significant interaction meant that the model identified a different temperature threshold between regions. The difficulty in pinpointing a threshold value was also found in Canada, where the temperature threshold for *I. scapularis* has been identified as a range, rather than a specific value [46]. Factors other than temperature, such as habitat suitability as driven by vegetation and host abundance, may differ between regions, in which case integrating biogeographic regions, as well as the country and years as random effects, serves as an overall proxy for habitat features that may affect their capacity to act as a shelter from extremes (e.g. type of forest floor covering). A difference in threshold could also relate to the genetic diversity of *I. ricinus* populations [47] if this has an impact on temperature-dependent interstadial development rates (i.e. if life-cycles are longer or shorter in different populations given the same temperature conditions), results in differences in daily per-capita mortality rates or affects host-seeking activity. While plasticity in host-seeking activity has been observed [48], precisely what drives observed differences is not clear.

Our results are corroborated through a comparison with the proportion of GBIF observations recorded above this threshold and Wilcoxon’s rank test, and are consistent with those reported in other relevant studies. Our thresholds values are similar to that identified for *I. scapularis* in Central and Eastern Canada [34] at 2800–3100 ADD > 0 °C, which is not unexpected based on the similarity of *I. scapularis* and *I. ricinus*. In our study area, the observed change in the position of the threshold is consistent with observations collated in the Boreal region of Norway [22], as well as in the Atlantic region of Norway [22, 23]. Furthermore, in Norway, Mysterud et al. showed that the rise in tick-borne disease incidence, while driven by diverse factors, was heavily affected by changes in vector abundance or distribution, as indicated by the similarity in patterns between pathogens that have different reservoirs [31]. Seasonality and spring warming may have played an important role as well [45]. In Sweden, surveys covering the period 1994–2010 showed that ticks appeared in North Sweden and that abundance had intensified in South and Central Sweden [49]. Studies in Norway (Atlantic region) and Sweden ([5, 23], respectively), identified a threshold growing season of 170 days and confirmed that the position of this threshold has moved northward in the respective study areas. Modelling approaches can also yield meaningful comparisons. Estrada-Peña et al. [50] assessed the role of climate in tick distribution over the period 1900–1999 using a presence-based model. The spatial patterns are consistent, even though this associative approach found a greater role for precipitation. It should be noted that the time periods covered are only partially coherent, and that the climate data sets are different. In Finland (Boreal region), the incidence of tick-borne diseases has increased over the past 25 years (e.g. [51]), but cases are found beyond our identified threshold. The authors of a study using a mechanistic approach concluded that the speed of tick development increased substantially, in the Boreal region in particular, but no specific climate threshold for tick persistence was identified [52]. More specific validation is hindered by differences in the methods or measurements used, but that also suggests that there is scope for further efforts in data compilation and exploration of mechanisms responsible for changes at various spatial scales.

Further investigation into the Atlantic and Continental portions of our study area may confirm the validity of the estimated thresholds. Various reports exist confirming the recently observed presence of *I. ricinus* along the Norwegian coast [22, 23, 53]. The rugged coastline in Norway is not well captured by the coarse climate data employed here, so comparing our threshold to local field studies is complicated (Additional file 1: Figures S6, S7, S8). Similarly, in the Continental region, the position of the threshold has no clear spatial position, likely in relation to the fact that most of it is above the threshold and largely suitable for *I. ricinus* (Additional file 1: Figures S6, S8). Nonetheless, we detected a positive latitudinal difference in threshold position between each year and the reference year over the period of interest (Additional file 1: Fig. SM7), indicating that temperature conditions for *I. ricinus* have improved over the study period. Qualitatively, the areas with annual DD > 0 °C above the respective threshold were larger in 2020 than at any time point we studied (Additional file 1: Figure SM8), although interannual fluctuations in ADD likely play a significant role in these patterns. In both the Atlantic and Continental regions, using a latitude-based indicator for change may not suitably capture altitudinal changes in tick presence or abundance that have been documented [17, 54].

Other environmental factors are important in providing ecological resources to *I. ricinus*, such as hosts and vegetation cover. The fact that *I. ricinus* occupies a very large area means that such resources will be very diverse across the distribution range, and ecological resources have also been changing across the continent over our study period. In Scandinavia, complex relationships exist with host distribution [31], a factor that has also changed substantially across the European continent over the past century [55], with the implication that further unravelling of the role of changes in environmental factors other than climate in limiting tick distribution may be warranted. While humidity may not be the limiting factor in the Boreal region, precipitation regimes may also affect tick habitat suitability [56].

The lack of long-term data series on tick abundance remains a significant challenge for studying the effects of environmental change, such as climate change, as long-term data are needed to understand better the mechanisms driving tick population dynamics. This limitation is also reflected in our validation, which utilised GBIF data, which may arise from a diversity of collection methods and sampling strategies and may not provide a complete representation of a species’ distribution [57]. Our results highlight the importance of standardised abundance and longitudinal data, as demonstrated by the VectorByte (https://www.vectorbyte.org/) platform and the VectAbundance database [58]. While promoting open data, these resources enhance modelling reliability and contribute to better public health preparedness by providing standardised, high-quality datasets that allow for more accurate analysis of vector population dynamics.

## Conclusions

We derived a temperature threshold from nymphal abundance of *Ixodes ricinus* that we projected onto historical temperature records. The results indicated that the position of the threshold has moved northward by about 400 km in the Boreal region since 1979. This result is consistent with the understanding of the biology of the tick, the potential effect of a change in climate on distribution and observations in the field. Our model combined nymphal abundance data collected over a large area in a robust fashion and climate reanalysis data. The results shed striking light on the changes in thermal suitability in the north of Europe and may bring support to many fine-scale studies of ticks or public health time series that have hypothesised effects of climate change as well as other environmental changes on tick abundance, pathogen prevalence and disease incidence.

## Supplementary Information

Additional file 1. 

## Data Availability

Data supporting the main conclusions of this study are included in the manuscript.
