# Supplementary material for: Northward expansion of the thermal limit for the tick Ixodes ricinus over the past 40 years
Source: Parasit Vectors. 2025 Nov 5;18:449. doi: 10.1186/s13071-025-07084-4 (PMC12590674; doi:10.1186/s13071-025-07084-4)
Supplement: Supplementary file 1 — Additional file 1. [file 13071_2025_7084_MOESM1_ESM.docx]

**Additional file of**

**Northward expansion of the thermal limit for the tick *Ixodes ricinus* over the past 40 years**


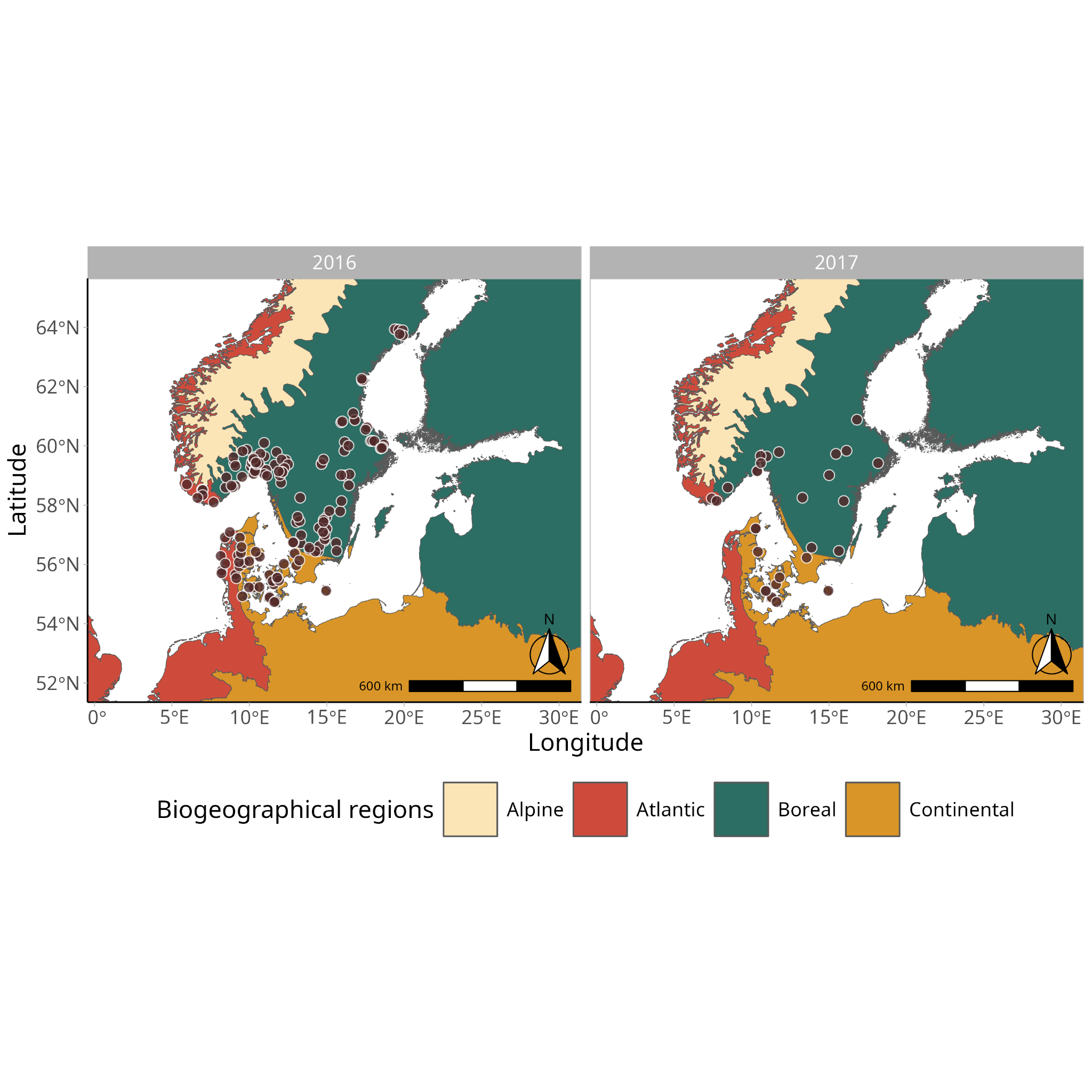
**Figure S1**: Biogeographical regions of Europe according to Cervellini et al., (2020) in the area of interest and the location (brown dots) of the locations sampled in 2016 and 2017.

**Table S2**: Contingency table of the observations broken down by country, year and biogeographical region.

| **Biogeographical Region** | **Country** | **2016** | **2017** |
| --- | --- | --- | --- |
| Atlantic | Denmark | 9 | 0 |
| Atlantic | Norway | 7 | 3 |
| Atlantic | Sweden | 0 | 0 |
| Boreal | Denmark | 0 | 0 |
| Boreal | Norway | 40 | 7 |
| Boreal | Sweden | 70 | 9 |
| Continental | Denmark | 28 | 10 |
| Continental | Norway | 0 | 0 |
| Continental | Sweden | 5 | 1 |

**S3-Description of the model selection**

We began the model selection process with a full generalized linear mixed-effects model (GLMM) using a Poisson distribution and a log link, which included both linear and quadratic terms for cumulative temperature (annual degree days above 0°C, hereafter ADD > 0°C), their interactions with biogeographical region (bgr), and random intercepts for *Country* and *Year*. The full model was specified as:

nymph abundance ~ ADD > 0°C * biogeographical regions + (ADD > 0°C)^2 * biogeographical regions + (1|Country) + (1|Year)

This model aimed to capture both linear and non-linear effects of temperature on nymph abundance, while allowing these effects to vary among biogeographical regions. However, initial model diagnostics revealed issues with convergence and unstable coefficient estimates, likely due to overparameterization, collinearity, or limited data in certain regional strata. Standard errors and test statistics were not defined (NaN), indicating that the model was overfitted and not suitable for inference.

To address this, we simplified the fixed-effects structure by sequentially removing higher-order terms and testing nested models using Akaike Information Criterion (AIC) and likelihood ratio tests. During this process, we retained random intercepts for *Country* and *Year* to account for spatial and temporal structure in the data. Including *Country* captures potential heterogeneity in sampling effort and habitat types among countries, while Year accounts for inter-annual variability in tick population dynamics. Both random effects were supported by improved model fit and meaningful variance estimates.

The selected reduced model included an interaction between linear GDD > 0°C and biogeographical region:

nymph abundance ~ ADD > 0°C * biogeographical regions + (1|Country) + (1|Year)

This model showed stable convergence and significant fixed effects for both temperature and region, as well as their interaction. Specifically, temperature was positively associated with nymph abundance in the Atlantic and Boreal regions, while the interaction term in the Continental region indicated a weak temperature effect at higher values of ADD > 0°C, but not significant.

Model comparison based on AIC and a likelihood ratio test indicated strong overdispersion in the Poisson model, a common issue when modelling ecological count data. The negative binomial version of the model provided a substantially better fit (ΔAIC > 5000; χ² = 5138, *p* < 0.001), and was therefore selected as the final model for inference.

We further evaluated the adequacy of the selected negative binomial model using the DHARMa diagnostic package. The nonparametric dispersion test showed no significant overdispersion, and the zero-inflation test also indicated no evidence of excess zeros. These results confirmed that the negative binomial model appropriately captured the structure of the observed nymph counts without requiring additional complexity.

Finally, the spatial autocorrelation of the GLMM residuals revealed a low but statistically significant positive autocorrelation ( Moran’s I observed = 0.144, expected = –0.005, p < 0.001). To account for this spatial structure, we tested the inclusion of the first two Moran’s Eigenvector Maps (MEM1 and MEM2) as fixed spatial covariates in the GLMM. However, their inclusion did not significantly improve model performance compared to the baseline model without MEMs (ΔAIC = 2.69; χ² = 1.31, df = 2, p = 0.52), and hence we kept the model formulation described in Eq. 1.


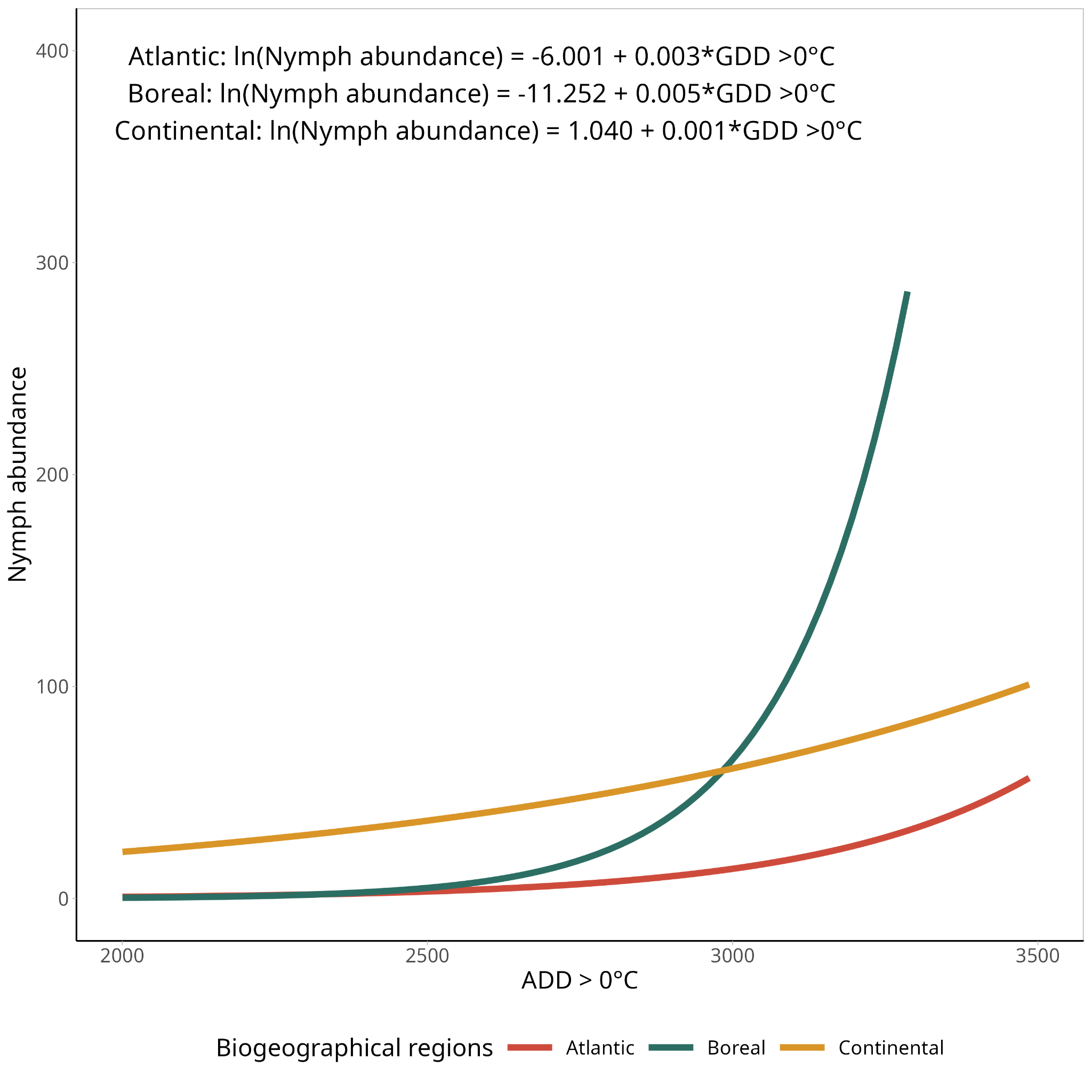
**Figure S4**: Relationships between the *Ixodes ricinus* nymph abundance and the annual degree days > 0°C (ADD) for each biogeographical region as estimated by the generalised linear mixed model defined in Eq. 1.
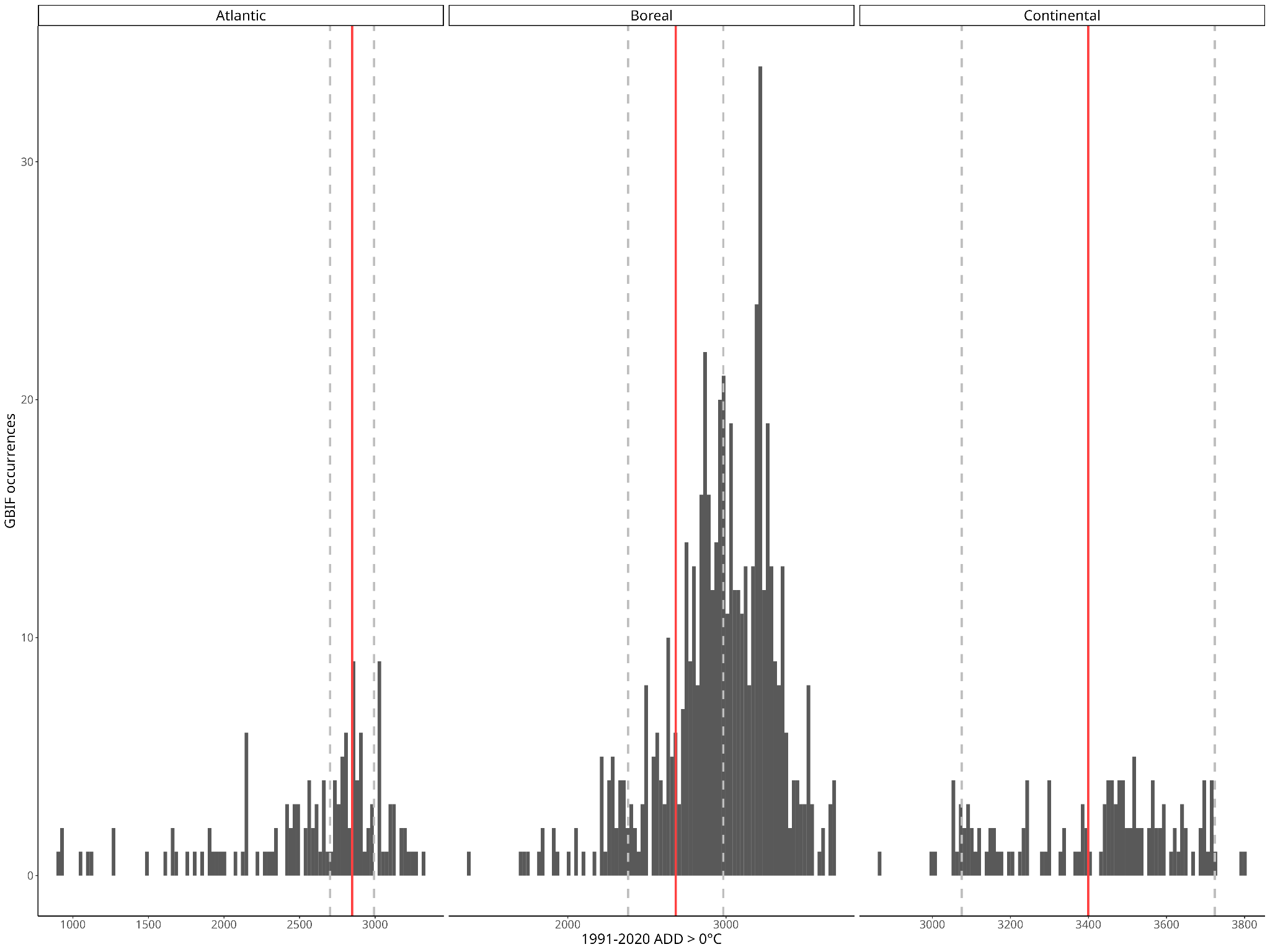
**Figure S5**: Distribution of GBIF occurrence over annual DD > 0 °C (histograms) and annual DD > 0 °C threshold (red line; dashed grey lines represent the confidence interval).

**
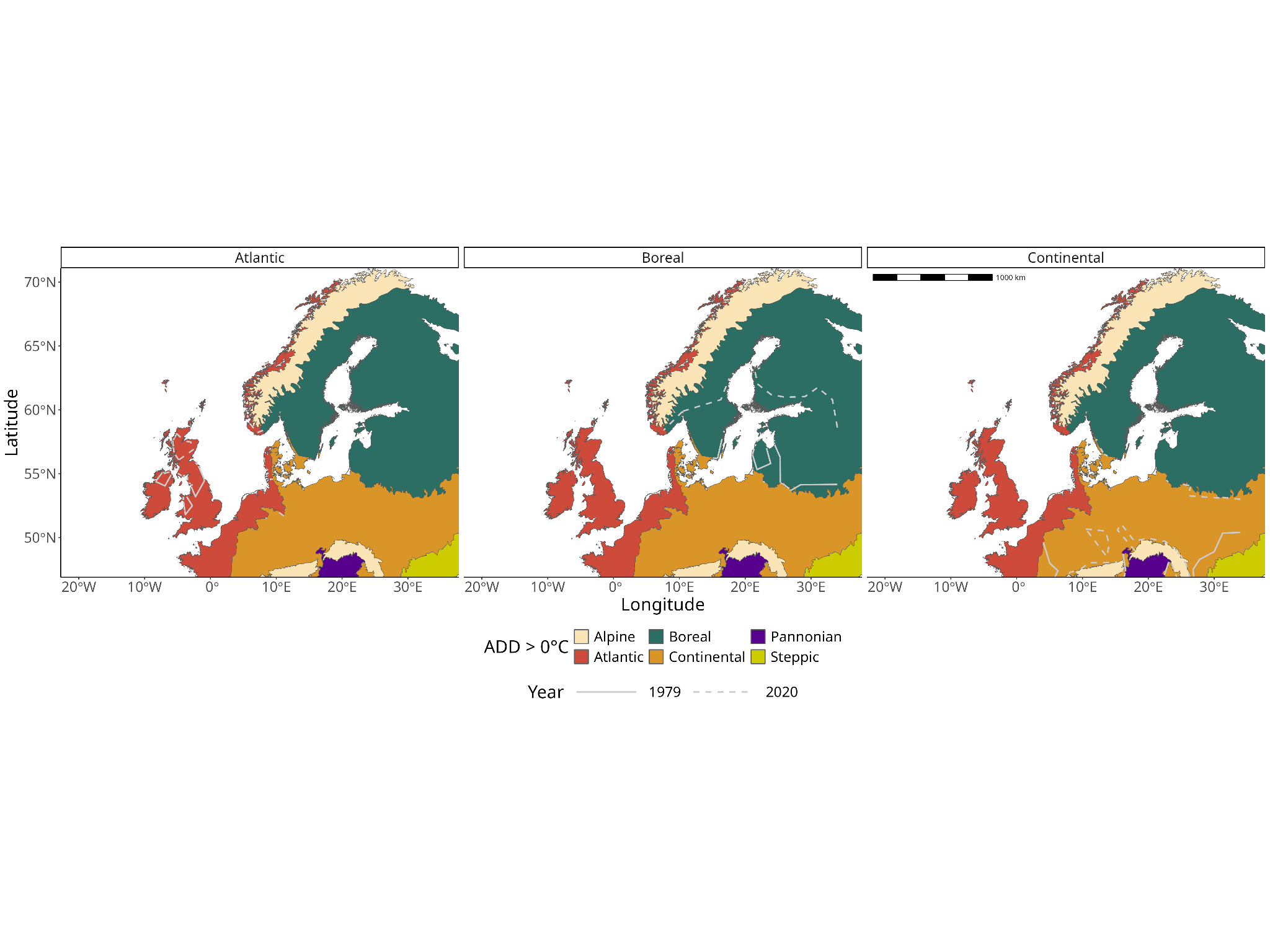
**

**Figure S6:** Mapped annual DD > 0 °C isolines thresholds across a larger portion of continental and northern Europe for the reference years 1979 and 2020.


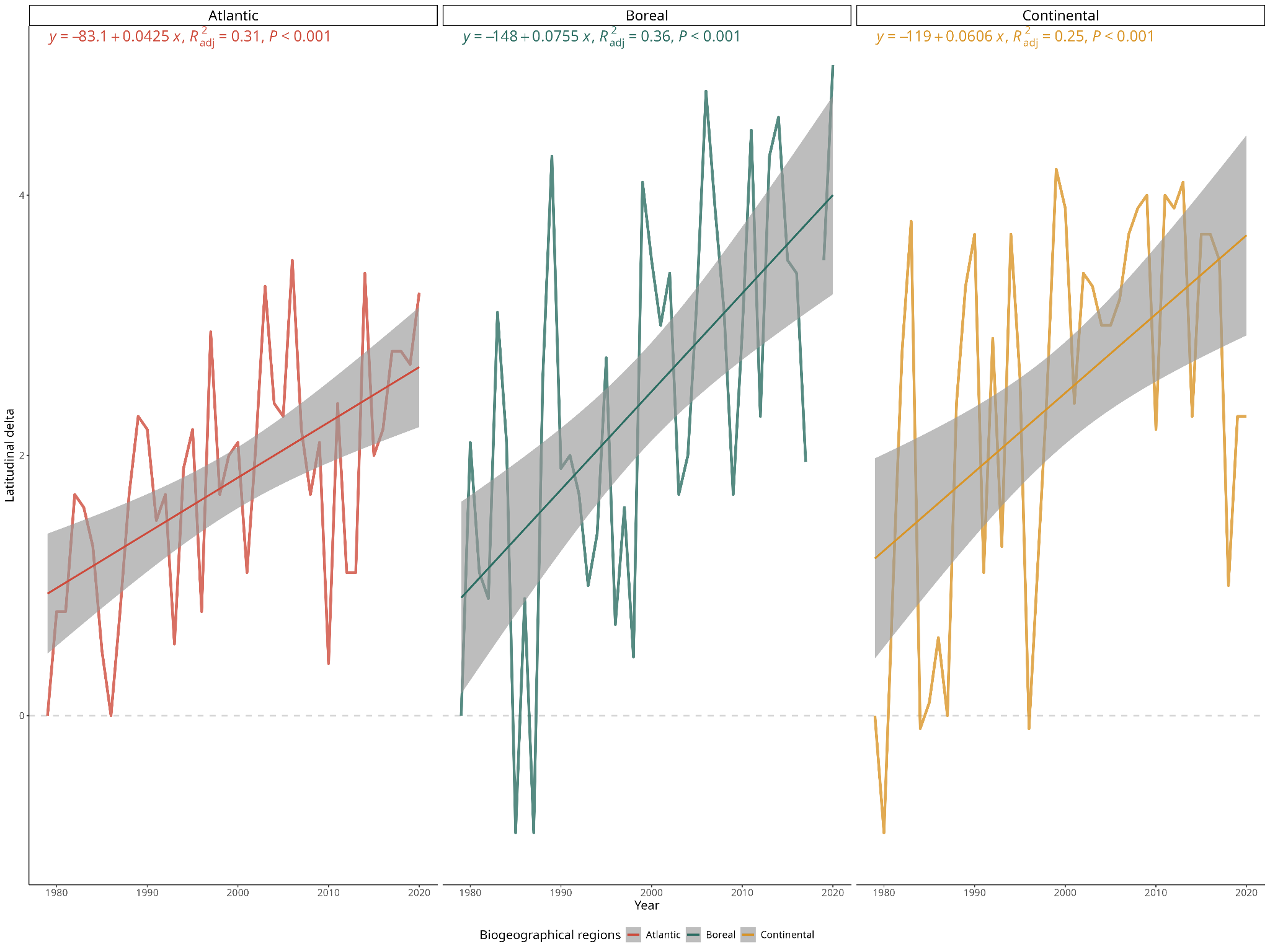


**Figure S7:** Variability of the Latitudinal delta between each year and the reference year 1979 in each biogeographical region.


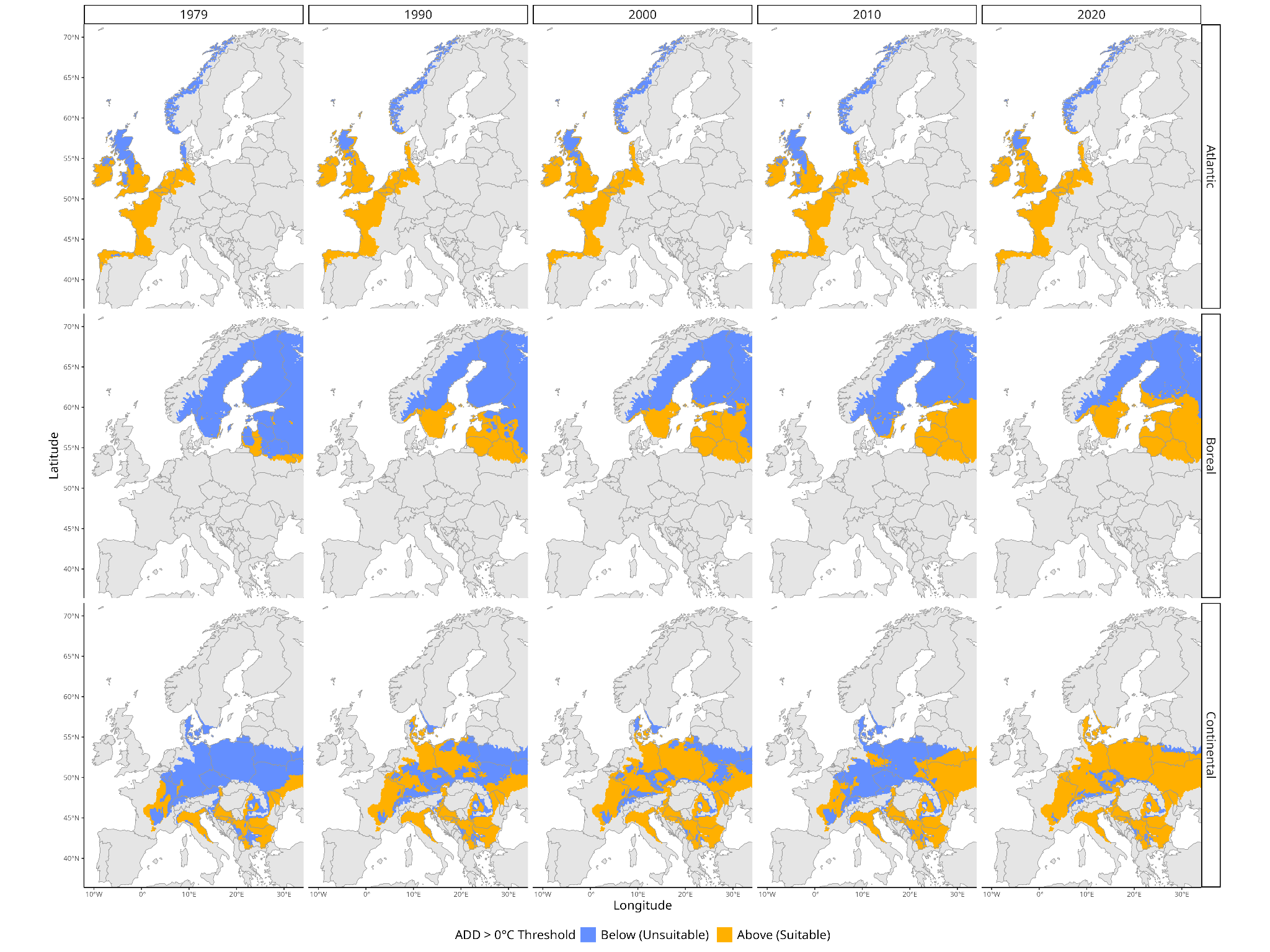


**Figure S8** Areas of Europe with annual Degree Days > 0°C values exceeding the thresholds for the three biogeographical regions (rows) and five reference years (columns): 1979, 1990, 2000, 2010, and 2020.
